# Epidemiological features and time-series analysis of influenza incidence in urban and rural areas of Shenyang, China, 2010–2018

**DOI:** 10.1017/S0950268820000151

**Published:** 2020-02-14

**Authors:** Ye Chen, Kunkun Leng, Ying Lu, Lihai Wen, Ying Qi, Wei Gao, Huijie Chen, Lina Bai, Xiangdong An, Baijun Sun, Ping Wang, Jing Dong

**Affiliations:** 1 Department of Infectious Disease, Shenyang center for Disease Control and Prevention, Shenyang 110031, Liaoning Province, PR China; 2 Department of Occupational and Environmental Health, School of Public Health, China Medical University, No.77 Puhe Road, Shenyang North New Area, Shenyang 110122, P.R. China

**Keywords:** Influenza strains, influenza-like illness, seasonal distinctions, surveillance

## Abstract

In recent years, there have been a significant influenza activity and emerging influenza strains in China, resulting in an increasing number of influenza virus infections and leading to public health concerns. The aims of this study were to identify the epidemiological and aetiological characteristics of influenza and establish seasonal autoregressive integrated moving average (SARIMA) models for forecasting the percentage of visits for influenza-like illness (ILI%) in urban and rural areas of Shenyang. Influenza surveillance data were obtained for ILI cases and influenza virus positivity from 18 sentinel hospitals. The SARIMA models were constructed to predict ILI% for January–December 2019. During 2010–2018, the influenza activity was higher in urban than in rural areas. The age distribution of ILI cases showed the highest rate in young children aged 0–4 years. Seasonal A/H3N2, influenza B virus and pandemic A/H1N1 continuously co-circulated in winter and spring seasons. In addition, the SARIMA (0, 1, 0) (0, 1, 2)_12_ model for the urban area and the SARIMA (1, 1, 1) (1, 1, 0)_12_ model for the rural area were appropriate for predicting influenza incidence. Our findings suggested that there were regional and seasonal distinctions of ILI activity in Shenyang. A co-epidemic pattern of influenza strains was evident in terms of seasonal influenza activity. Young children were more susceptible to influenza virus infection than adults. These results provide a reference for future influenza prevention and control strategies in the study area.

## Introduction

Influenza is an acute respiratory infectious disease with a high rate of morbidity and mortality [1–3]. Extensive studies have been performed to identify epidemiological and aetiological characteristics of influenza in order to explore a scientific strategy for its prevention and control [4–6]. Relevant literature indicates that influenza A and B viruses undergo continuous changes due to antigenic shift or drift and consequently change into new subtypes [7, 8]. Viral drift and shift are the reasons for seasonal epidemics or unpredictable pandemics, which lead to enormous financial losses and disease burden to the public. Recently, the burden of influenza among high-risk groups in the USA and other countries has been well described [9, 10]. Influenza pandemics and epidemics are associated with the spread of novel or re-emerging viruses which primarily originate from homologous influenza viruses. Hence, it is particularly important to explore epidemiological characteristics and circulating virus strains of influenza and identify vulnerable groups among the population.

In 2005, the National Influenza Surveillance System in China was established to monitor the epidemic pattern and intensity of influenza. In addition, an influenza surveillance system was formed through the combination of 18 sentinel hospitals in urban and rural areas of Shenyang, which can constantly monitor influenza-like illness (ILI). A sentinel laboratory network, including three national sentinel hospitals, in Shenyang is responsible for enhancing the surveillance system to comprehensively monitor and understand influenza activity and transmission and the circulating strains of influenza virus. Shenyang, the capital city of Liaoning Province, is located in northeast China and has clear seasonal variability. It administers 10 municipal districts in the urban area and four counties in the surrounding rural areas. Differences in demographic composition, population mobility, economic levels and medical services between these urban and rural areas may influence influenza epidemics, severity and transmission. Therefore, it is necessary to illustrate the geographical difference in the distribution of ILI activity between urban and rural areas in order to guide the regionalisation of surveillance.

Since the 2009 influenza pandemic, influenza surveillance has been considered a public health event of international concern. The surveillance scheme is carried out constantly with the objective of characterizing influenza activity to inform public health prevention and control activities. Although the influenza surveillance system enhanced our effectiveness in understanding influenza epidemics, severity and virology, the time-series models for the prediction of influenza activity in Shenyang were not well established, particularly in light of the emerging influenza pandemic in recent years. A widely used prediction model, the autoregressive integrated moving average (ARIMA) model, has been applied to predict the incidence of various infectious diseases, such as malaria, haemorrhagic fever and hand–foot–mouth disease [11–13]. Based on time-series data, the ARIMA model takes into account changing trends, periodicity and random disturbance, which gives it a capacity for short-term forecasting [14]. In addition, the seasonal ARIMA (SARIMA) model can give temporal trends of seasonality if distinct seasonality exists in time-series data [11]. Therefore, it was speculated that the SARIMA model could be used to predict the incidence of influenza in Shenyang, which could provide reference data for future influenza prevention and control strategies.

The purposes of this study were to explore the regional and seasonal distinctions of ILI activity, circulating strains of influenza virus and vulnerable groups among the population in Shenyang during 2010–2018, and to establish time-series models in urban and rural areas for short-term prediction.

## Methods

### Data sources

The influenza surveillance system of Shenyang is composed of 18 sentinel hospitals, including 14 hospitals in charge of 10 municipal districts of the urban area and four hospitals located in rural areas under the jurisdiction of Shenyang. The data of outpatients visiting hospitals were derived from the departments of paediatrics, respiratory and emergency. The ILI cases were identified by the sentinel general practitioners. The numbers of total visits and ILI cases were reported to the Shenyang Municipal Center for Disease Control and Prevention. The outpatients were classified by age into five groups: 0–4, 5–14, 15–24, 25–59 and ≥60 years. The ILI% was defined as the total number of ILI cases among the total number of outpatients visiting hospitals.

### Case definition and laboratory testing

The ILI was defined as a person with a sudden onset of fever (≥38 °C), chills, cough and/or sore throat, a generalised feeling of weakness and pain in the muscles, together with varying degrees of soreness in the head and abdomen. Approximately 30–45 specimens (nasopharyngeal swabs) of ILI cases from three national sentinel hospitals were transferred to the Shenyang Municipal Center for Disease Control and Prevention. Influenza-positive cases were confirmed by real-time reverse transcription PCR. Laboratory-confirmed influenza was further analysed to determine the subtypes of influenza virus.

### Establishment of the SARIMA model

The ARIMA model is based on the sequentially lagged relationships existing in the time-series data [15]. If the time series showed no seasonality, the ARIMA model was appropriate for predicting ILI%. However, the SARIMA model was considered more suitable for forecasting if there were obvious seasonal characteristics in the data. The SARIMA model can be expressed as SARIMA (*p*, *d*, *q*) (*P*, *D*, *Q*) *s*. Letters *p*, *d* and *q* represent the order of autoregression, the degree of difference and the order of moving average, respectively. Letters *P*, *D* and *Q* represent seasonal autoregression, seasonal integration and seasonal moving average, respectively, and *s* is the length of the seasonal period.

**Fig. 1.**
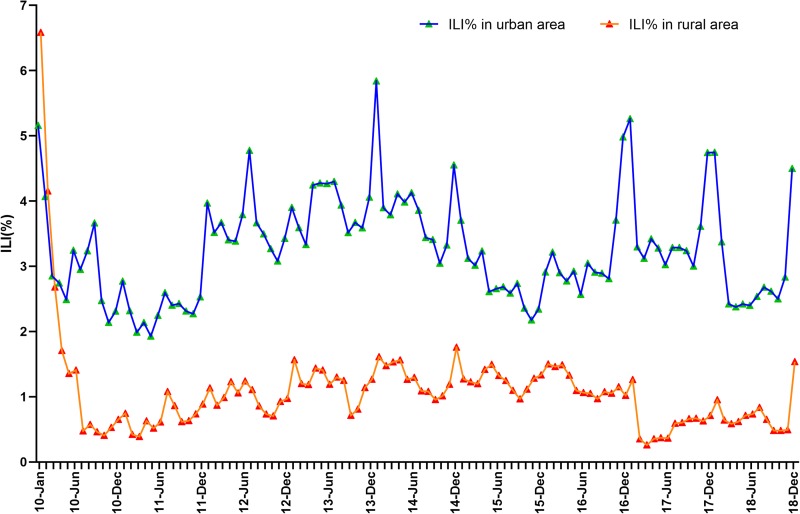
The monthly percentage of visits for influenza-like illness (ILI%) of urban and rural areas in Shenyang, 2010–2018.

In this study, the establishment of the SARIMA model was divided into three steps: identification, estimation and diagnosis. First, time-series plots of the monthly ILI% were drawn to check stationarity and seasonality. The model (*s* = 12) was constructed, and the autoregressive and moving average parameters were confirmed by depicting the auto-correlation function (ACF) and partial auto-correlation function (PACF) of model residuals. Second, several alternative models were constructed and the Ljung–Box *Q* test was used to estimate the ACF and PACF of residuals. If the residuals were equal to white noise (*P* > 0.05), the optimal SARIMA model was regarded as the model with the lowest value of the Bayesian Information Criterion (BIC). Finally, this model was applied to forecast the monthly ILI% for January–December 2019. The mean absolute percentage error (MAPE), root mean square error (RMSE) and stationary *R*^2^ (coefficient of determination) were used to estimate the goodness-of-fit and effectiveness of the model. In addition, during the modelling process, it was found that the SARIMA model of the rural area was always unstable and ineffective, resulting in abnormal and invalid predicted values for ILI% of the rural areas. This was possibly due to the higher ILI% for January–April 2010 compared with other years (Fig. 1). Hence, the ILI% of 2010 was removed from the time series in view of the stability of the prediction model.

### Ethical statement

The influenza surveillance is a governmental public health task in the charge of the Shenyang Municipal Center for Disease Control and Prevention and sentinel hospitals. Therefore, an ethical review by an ethics committee was not required. Verbal consent was obtained from each ILI case previous to specimen collection. Personal identifiable information of outpatients, including name, address, phone number and other health conditions, was not disclosed to ensure personal individual privacy.

### Statistical analysis

The *χ*^2^ test was used to test the differences in categorical groups. Spearman correlation analysis was applied to determine the relationship of ILI% in urban and rural areas of Shenyang. Excel 2010 was used to sort the data, and SPSS 21.0 (SPSS Inc., Chicago, IL, USA) was applied to analyse data and establish the SARIMA model. A two-sided *P*-value < 0.05 was considered significant.

## Results

### Influenza surveillance in urban and rural areas for 2010–2018

The time-series plots showed that ILI activity during the study period had obvious seasonality and periodicity in urban and rural areas (Fig. 1). Generally, the active phase of ILI commenced in winter, and peaked in December–January every year. In comparison, ILI activity in autumn was at a relatively low level and reached a minimum during September–October every year. However, the ILI% showed short-term upward trends during each summer in 2010–2018. We noted that ILI activity in the rural areas for January–April 2010 was at a high level, with much higher ILI% than in other years, and then returned to the inter-seasonal level by May 2010. It was also found that the ILI% of 2010–2018 between urban and rural areas were significantly correlated (*r* = 0.413, *P* < 0.001), but the epidemic intensity of ILI activity was higher in urban than in rural areas.

During 2010–2018, of the 752 695 reported ILI cases, 736 255 (97.82%) were reported by urban hospitals and 16 440 (2.18%) were from the rural areas. The total number of visiting outpatients (24 159 315) reported by 18 sentinel hospitals during 2010–2018 was over 30 times the number of ILI cases in this period, thus giving a crude ILI% of 3.12% in Shenyang (Table 1). Overall, the timing and amount of ILI activity varied across regions, seasons and years. For example, ILI activity showed significant regional differences with ILI% of 3.27% and 1.00% in urban and rural areas, respectively. Winter had an increasing number of ILI cases, and this was more than in other seasons. The ILI% increased from 2011 followed by a period of increase of ILI cases during 2012–2014. Additionally, the proportion of ILI cases in age and season groups significantly differed between urban and rural areas (*P* < 0.001). Children aged 0–4 years had the highest proportion of ILI cases (46.90% for urban and 49.43% for rural), followed by corresponding values for 5–14 years of 23.09% and 23.52%, and for ≥15 years of 30.02% and 27.04% (Table 2).

**Table 1.** Distribution of the influenza-like illness (ILI) cases by age and season group in urban and rural areas of Shenyang, 2010–2018

	0–4	5–14	15–24	25–59	≥60			
			
Characteristic	No. of ILI	No. of ILI	No. of ILI	No. of ILI	No. of ILI	Total	No. of outpatients	ILI%
Year								
2010	25 056 (41.35%)^a^	10 781 (17.79%)	6829 (11.27%)	12 283 (20.27%)	5645 (9.31%)	60 594	2 006 038	3.02
2011	21 418 (46.13%)	8007 (17.25%)	4691 (10.10%)	8442 (18.18%)	3869 (8.33%)	46 427	2 077 450	2.23
2012	36 508 (48.99%)	17 449 (23.42%)	5985 (8.03%)	9505 (12.76%)	5072 (6.81%)	74 519	2 150 585	3.47
2013	37 723 (43.71%)	19 687 (22.81%)	7589 (8.79%)	14 977 (17.35%)	6330 (7.33%)	86 306	2 301 073	3.75
2014	38 383 (41.96%)	23 878 (26.10%)	5882 (6.43%)	17 432 (19.06%)	5896 (6.45%)	91 471	2 386 773	3.83
2015	38 142 (50.26%)	20 397 (26.88%)	3601 (4.75%)	9982 (13.15%)	3767 (4.96%)	75 889	2 870 874	2.64
2016	48 238 (48.56%)	23 766 (23.93%)	5205 (5.24%)	15 936 (16.04%)	6188 (6.23%)	99 333	3 252 130	3.05
2017	61 740 (53.76%)	25 197 (21.94%)	5054 (4.40%)	16 221 (14.12%)	6631 (5.77%)	114 843	3 432 701	3.35
2018	46 201 (44.72%)	24 737 (23.94%)	6014 (5.82%)	18 306 (17.72%)	8055 (7.80%)	103 313	3 681 691	2.81
Seasons								
Spring (Mar–May)	80 777 (45.77%)	38 925 (22.05%)	13 248 (7.51%)	30 470 (17.26%)	13 082 (7.41%)	176 502	6 050 491	2.92
Summer (Jun–Aug)	90 386 (49.90%)	43 577 (24.06%)	10 480 (5.78%)	25 497 (14.08%)	11 192 (6.18%)	181 132	6 058 426	2.99
Autumn (Sep–Nov)	80 945 (49.57%)	37 976 (23.26%)	10 283 (6.30%)	23 285 (14.26%)	10 793 (6.61%)	163 282	5 769 136	2.83
Winter (Dec–Feb)	101 301 (43.71%)	53 421 (23.05%)	16 839 (7.26%)	43 832 (18.91%)	16 386 (7.07%)	231 779	6 281 262	3.69
Region								
Urban area	345 282 (46.90%)	170 032 (23.09%)	49 157 (6.68%)	121 816 (16.55%)	49 968 (6.79%)	736 255	22 520 715	3.27
Rural area	8127 (49.43%)	3867 (23.52%)	1693(10.30%)	1268(7.71%)	1485 (9.03%)	16 440	1 638 600	1.00
Total	353 409 (46.95%)	173 899 (23.10%)	50 850 (6.76%)	123 084 (16.35%)	51 453 (6.84%)	752 695	24 159 315	3.12

ILI%, the percentage of visits for ILI (total number of ILI cases/total number of visiting outpatients).

a Indicated the constituent ratio of ILI by age group.

**Table 2.** Demographic characteristics of the influenza-like illness (ILI) cases by the influenza sentinel surveillance system of Shenyang, 2010–2018

Characteristic	Overall (%)	Urban (%)	Rural (%)	*P*-value
Age, year				<0.001
0–4	353 409 (46.95%)^a^	345 282 (46.90%)	8127 (49.43%)	
5–14	173 899 (23.10%)	170 032 (23.09%)	3867 (23.52%)	
15–24	50 850 (6.76%)	49 157 (6.68%)	1693 (10.30%)	
25–59	123 084 (16.35%)	121 816 (16.55%)	1268 (7.71%)	
≥60	51 453 (6.84%)	49 968 (6.79%)	1485 (9.03%)	
Seasons				<0.001
Spring (Mar–May)	176 502 (23.45%)	172 351 (23.41%)	4151 (25.25%)	
Summer (Jun–Aug)	181 132 (24.07%)	177 271 (24.08%)	3861 (23.49%)	
Autumn (Sep–Nov)	163 282 (21.69%)	160 049 (21.74%)	3233 (19.66%)	
Winter (Dec–Feb)	231 779 (30.79%)	226 584 (30.77%)	5195 (31.60%)	
Total	752 695 (100%)^b^	736 255 (97.82%)	16 440 (2.18%)	

a Indicated the constituent ratio of ILI by age and season group.

b Indicated the constituent ratio of ILI by region.

### Laboratory-confirmed influenza virus for 2010–2018

The 17 239 specimens provided by three national sentinel hospitals were tested during 2010–2018. Of the total positive specimens for influenza virus (*n* = 1786), more than half were influenza A virus (60.70%; *n* = 1084). Influenza type B infections accounted for 37.40% (*n* = 668), and only 1.90% (*n* = 34) were of hybrid type. Of influenza A virus, nearly all were seasonal A/H3N2 (*n* = 470) and pandemic A/H1N1 (*n* = 609). In addition, pandemic A/H1N1 and influenza B virus circulated sporadically during winter and spring seasons of the study period. Seasonal A/H3N2 viruses were reported annually in autumn and winter, and seasonal A/H3N2 circulated intensively in the 2015–2016 influenza seasons, replacing pandemic A/H1N1 (Table 3).

**Table 3.** Number and rate of laboratory-confirmed influenza virus strain by season, during 2010–2018

Seasons	Specimens	No. of positive	Positive rate (%)	Influenza A virus			Influenza B virus	Hybrid type
				H1N1	H3N2	pdm H1N1		
Spring (Mar–May)	3486	489	14.03	2 (0.41%)^a^	41 (8.38%)	124 (25.36%)	319 (65.24%)	3 (0.61%)
Summer (Jun–Aug)	1973	29	1.47	0 (0%)	16 (55.17%)	0 (0%)	2 (6.90%)	11 (37.93%)
Autumn (Sep–Nov)	4870	115	2.36	0 (0%)	68 (59.13%)	21 (18.26%)	12 (10.43%)	14 (12.17%)
Winter (Dec–Feb)	6910	1153	16.69	3 (0.26%)	345 (29.92%)	464 (40.24%)	335 (29.05%)	6 (0.52%)
Total	17 239	1786	10.36	5 (0.28%)	470 (26.32%)	609 (34.10%)	668 (37.40%)	34 (1.90%)

a Indicated the constituent ratio of influenza virus.

**Fig. 2.**
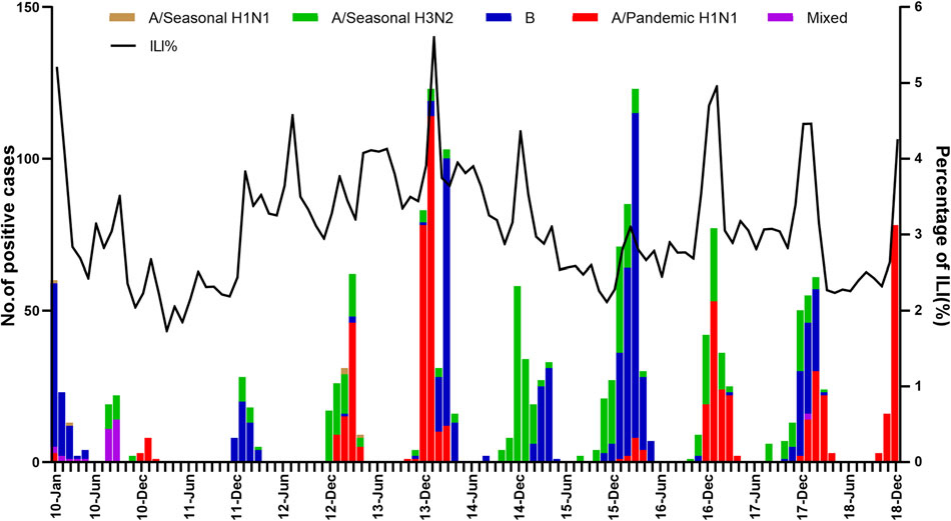
Monthly distribution of laboratory-confirmed influenza cases and ILI% of Shenyang during 2010–2018.

Laboratory-confirmed influenza cases were mainly distributed in winter in accordance with ILI activity. A co-epidemic pattern of pandemic A/H1N1 and seasonal A/H3N2 and B virus emerged from 2013 and the dominant strain changed each year. The number of influenza-positive cases increased sharply during 2011–2013. Pandemic A/H1N1 was the predominant strain of influenza viruses in 2013, 2017 and 2018. Seasonal A/H3N2 co-circulated with influenza B virus in winter and spring seasons of most years (Fig. 2).

### Time-series analysis of ILI% of urban and rural areas

The ILI% in the rural area was extraordinarily higher in early 2010 than in other years (Fig. 1). Preliminary modelling results of the rural area showed an unstable and ineffective SARIMA model with abnormal and invalid predicted values. The 2009 H1N1 pandemic was officially over on 10 August 2010 [16], and consequently, the ILI activity of 2010 may be associated with this pandemic. We removed the ILI% of 2010 from the time-series data of urban and rural areas to ensure the validity and stability of the models.

**Fig. 3.**
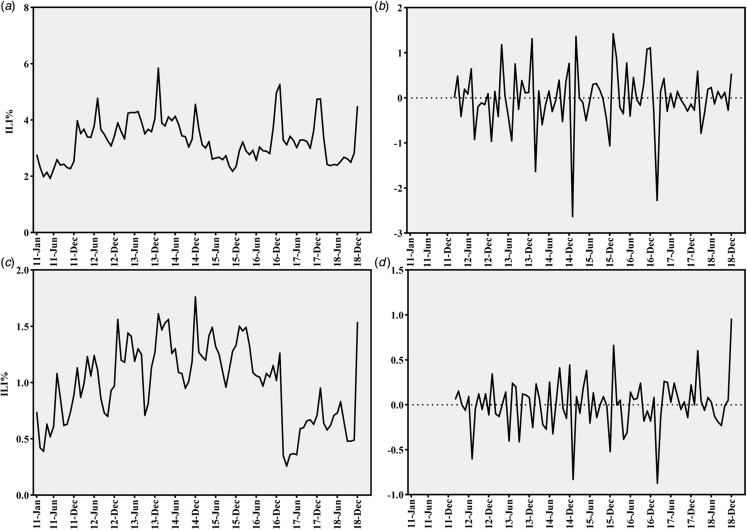
The time series for monthly ILI% at non-seasonal difference and (or) seasonal difference during 2011–2018. (a) Original data of urban area; (b) data of urban area at first-order non-seasonal difference (*d* = 1) and first-order seasonal difference (*D* = 1); (c) original data of rural area; (d) data of rural area at *d* = 1 and *D* = 1.

**Fig. 4.**
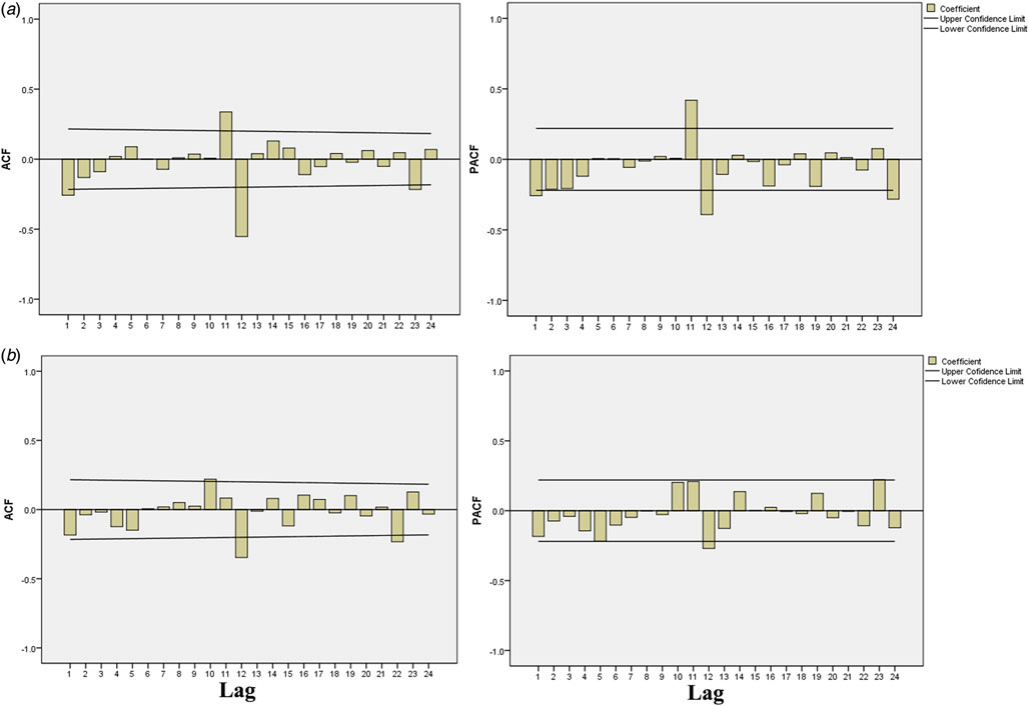
Auto-correlation function (ACF) and partial auto-correlation function (PACF) graphs of monthly ILI% at non-seasonal difference and (or) seasonal difference. (a) ACF and PACF graphs for urban area at *d* = 1, *D* = 1. (b) ACF and PACF graphs for rural area at *d* = 1, *D* = 1.

The SARIMA models for urban and rural areas were constructed separately using the following steps. First, the time-series plot of the urban area fluctuated around zero after first-order non-seasonal difference (*d* = 1) and first-order seasonal difference (*D* = 1) were calculated (Fig. 3b). Then, the ACF and PACF graphs were used to determine the model parameters. The ACF and PACF fell around zero within 95% confidence limits (CIs) except at the lags of 1, 12 and 24 (Fig. 4a), which revealed that this time series could be used to establish SARIMA (*p*, 1, *q*) (*P*, 1, *Q*)_12_. Several alternative models were constructed and then the Ljung–Box *Q* test and normalised BIC were used to determine the most appropriate one. Finally, we concluded that the SARIMA (0, 1, 0) (0, 1, 2)_12_ with BIC (−1.218), RMSE (0.516), MAPE (10.597) and stationary *R*^2^ (0.410) could be the perfect model for the urban area (Table 4). The SARIMA model for the rural area was similarly established according to the steps above. Therefore, we believe that SARIMA (1, 1, 1) (1, 1, 0)_12_ was suitable for the rural area.

**Table 4.** Goodness of statistics for SARIMA models

Model	Goodness-of-fit statistics				Ljung–Box *Q*		
	Stationary *R*^2^	RMSE	MAPE	BIC	Statistics	DF	*P*-value
SARIMA (0, 1, 0) (0, 1, 2)_12_ for urban area	0.410	0.516	10.597	−1.218	13.260	17	0.654
SARIMA (1, 1, 1) (1, 1, 0)_12_ for rural area	0.234	0.247	19.414	−2.636	9.915	15	0.825

Stationary *R*^2^, the coefficient of determination; RMSE, root mean square error; MAPE, mean absolute percentage error; BIC, Bayesian Information Criterion.

**Fig. 5.**
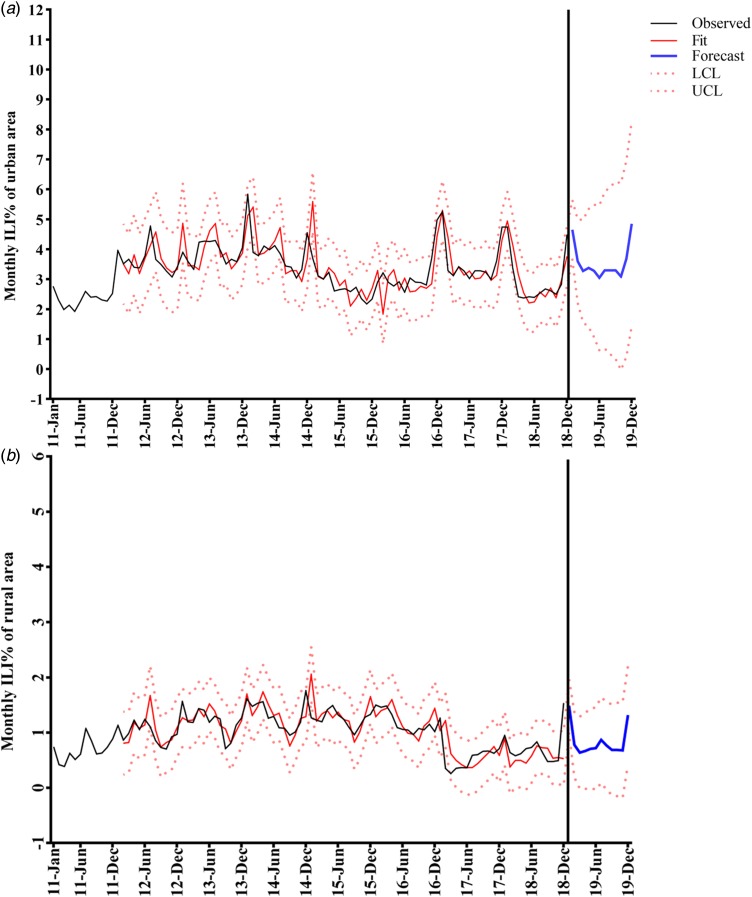
Time-series plots for predicted values of monthly ILI% by SARIMA model during 2011–2019. (a) Urban area. (b) Rural area. Dotted lines indicate the 95% confidence intervals (CIs) (UCL: upper limit of 95% CI; LCL: lower limit of 95% CI).

We applied SARIMA (0, 1, 0) (0, 1, 2)_12_ and SARIMA (1, 1, 1) (1, 1, 0)_12_ to forecast ILI% of urban and rural areas for January–December 2019. The fitting and forecasting results are shown in Fig 5. Compared with observed data of 2010–2018, the fitted values were consistent with the fluctuation trend of the actual values, which suggested a favourable goodness-of-fit.

## Discussion

This study presented findings from a retrospective analysis of influenza surveillance data collected under the influenza surveillance system of Shenyang during 2010–2018. The available data were utilised to analyse epidemiological and virological characteristics of influenza activity. Due to many differences between urban and rural areas, including demographic composition, economic levels and medical services, population mobility and living conditions, the data were divided into two main categories: urban and rural areas.

Although the fluctuation trends of ILI% of urban and rural areas were similar, the intensity of ILI activity was higher in urban than in rural areas. The result indicated that the spread and occurrence of influenza may be more prevalent in urban area. Generally, ILI surveillance was performed by general hospitals or urban paediatric hospitals. In the urban area, on the one hand, a continuous influx of migrants may bring about the emergence of influenza virus infections or unconfirmed ILI cases, which drove the spread of influenza among the population. On the other hand, there were large numbers of sentinel hospitals to facilitate communication between patients and physicians. Given a full coverage by the 14 sentinel hospitals of all the municipal districts in the urban area, we believe that the surveillance data of sentinel hospitals represented influenza activity in the urban area. With regard to the rural area, people who live in villages and towns generally visit primary hospitals or village clinics when developing ILI symptoms [17]. In addition, some people with suspected ILI symptoms were more likely to purchase medicine at pharmacies nearby instead of having a medical consultation or visiting a doctor. The reported data from the rural area probably represent only a small proportion of all ILI cases. The true incidence of ILI was likely to be underestimated in the rural area, especially in view of the poor transportation accessibility in these regions. Therefore, influenza surveillance of the rural area should be conducted comprehensively in the whole population through primary hospitals, village clinics and sentinel hospitals.

In this study, children aged ⩽4 years accounted for nearly half of all ILI cases. Similarly, previous studies indicated that influenza infections were most frequent in children aged <5 years [18, 19]. Influenza viruses can be transmitted by respiratory droplets and direct contact with an infected person or fomites. Infants and young children may be at a high risk of influenza virus infection [10, 20, 21]. Previous studies also confirmed that influenza virus and other pathogens, such as respiratory syncytial viruses (RSV), rhinoviruses and enteroviruses, might be more sensitive to immunocompromised or immunodeficient infants and young children, leading to concomitant or consecutive infection [21–24]. Our results highlighted that a very large amount of ILI cases emerged in younger age groups, which could provide evidence for the increasing literature confirming the population susceptible to influenza viruses. In addition, there may be differences in the rate and frequency of visiting medical institutions between children and adults. Children with ILI symptoms may be more prone to visit doctors than adults, resulting in a higher hospital visiting rate. Our study warrants further collection of more influenza data regarding visiting rates among different age groups.

Our results showed that the ILI% of urban and rural areas were higher in winter, consistent with laboratory-confirmed influenza cases. However, it should be noted that the ILI% showed short-term upward trends during each summer. This suggested that a considerable proportion of outpatients with ILI symptoms in summer might be caused by other pathogens, such as RSV, adenoviruses and parainfluenza 1, 2 and 3 viruses [25, 26]. Additionally, with a lack of laboratory confirmation of ILI, physicians have difficulty identifying influenza virus infection using their judgement of typical symptoms including sudden onset of fever, cough, sore throat and general malaise [24]. Furthermore, the influenza activity of the 2015–2016 influenza season was relatively more intense, with more influenza-positive cases than other seasons (except for 2013–2014), whereas the ILI% in 2015–2016 was lower than in other seasons. We speculate that the pathogen spectrum of ILI symptoms changed in 2015. The previous study indicated that approximately 60% of ILI cases were caused by non-influenza viruses [26]. So, compared to other influenza seasons, there might be lower infections of other respiratory pathogens involved in ILI symptoms in the 2015–2016 influenza season. Consequently, laboratory confirmation of various respiratory pathogens may be critical for influenza surveillance and prevention.

The seasonal patterns of ILI and circulating strains in Shenyang were similar to those of other regions in northern China [27–29]; however, the virological characteristics of influenza virus differed from those in southern China where an annual cycle with two peaks in summer and winter was pervasive [18, 30]. This discrepancy may be due to climate or meteorological factors. For example, lower humidity benefits virus survival and movement, and lower temperature in winter increases indoor activities and promotes the transmission of aerosol virus indoors, which appear to be related to influenza seasonal epidemics [31]. Previous studies also indicated that seasonal features of influenza viruses were associated with the local rainy season across the subtropical and tropical climates [32]. Shenyang is located in the temperate region with lower humidity and temperature. The impact of environmental and climatic factors on influenza epidemics has rarely been reported in Shenyang. Therefore, more epidemiological studies should be implemented to explore the factors affecting influenza activity in Shenyang, especially meteorological factors.

Laboratory findings indicated that pandemic A/H1N1, seasonal A/H3N2 and influenza B virus were major circulating strains of influenza virus in Shenyang. Seasonal A/H3N2 co-circulated with influenza B virus in the winter–spring epidemic season of most years. In total, influenza B virus accounted for the maximum number of laboratory-confirmed influenza cases, followed by pandemic A/H1N1 and seasonal A/H3N2. These results corresponded with the facts that a co-epidemic pattern of these three strains of influenza virus has formed in China since 2009, with seasonal A/H3N2 predominantly circulating in the south of China while influenza B virus and pandemic A/H1N1 are more frequent in northern China [4, 18, 19, 33–35]. It is noteworthy that the number of influenza-positive cases was much lower in 2011 and 2012 than other years. Also, the pandemic A/H1N1 became the dominant strain from the winter of 2013 and has prevailed in the winter–spring period since then, particularly in 2018. We speculate that the substantial increase of pandemic A/H1N1 was predominately driven by the loss of protective antibodies obtained from the 2009 influenza season. The data of laboratory-confirmed influenza in 2018 suggested that we should focus on the next possible pandemic of pandemic A/H1N1 to inform public health responses.

It has been proven that the SARIMA model is applicable to predict the incidence of infectious disease [11–13, 36]. However, this model may only be feasible for short-term prediction of influenza incidence owing to the increased relative bias of predicted values. We utilised the monthly ILI% of urban and rural areas in Shenyang for 2011–2018 and established SARIMA (0, 1, 0) (0, 1, 2)_12_ for the urban area and SARIMA (1, 1, 1) (1, 1, 0)_12_ for the rural area. We found that the goodness-of-fit index, including stationary *R*^2^ values, MAPE and RMSE, showed relatively reliable and high validity in comparison to other studies [13, 33, 36]. The fitted values for 2011–2018 showed seasonal peaks, coinciding with observed ILI%. We believe that these two models could be appropriate to predict ILI% for 2019 in urban and rural areas of Shenyang. Although predicted values can provide reference data for short-term incidence of influenza, the decisions and strategies for influenza prevention and control should comprehensively consider multiple influencing factors, such as the accuracy of monitoring data, pathogenic variation, geographical location and vaccination.

There are some limitations to our study. First, the emphasis of influenza surveillance was primarily in the urban rather than the rural area, and so was likely to underestimate the true ILI% or ILI activity in the rural area. Second, laboratory testing was not carried out in rural sentinel hospitals, which should be accounted for in future influenza surveillance. Finally, meteorological factors, such as temperature and relative humidity, were not considered.

In summary, differences in regional distinctions, seasonality and age distribution of ILI activity in Shenyang were identified. Our findings suggested that a co-epidemic pattern of influenza strains was evident in terms of seasonal influenza activity. In addition, influenza viruses were more sensitive to young children than adults. These results could provide a reference for future influenza prevention and control strategies in the study area.
